# Impact of Obesity on Outcomes of Gender-Affirming Mastectomies: A Single-Surgeon Experience

**DOI:** 10.3390/jcm14145092

**Published:** 2025-07-17

**Authors:** Yoram Wolf, Dvir Gilboa, Ron Skorochod

**Affiliations:** 1 Hillel Yaffe Medical Center, Hadera 38100, Israel; yoramw@hymc.gov.il; 2 Rappaport Faculty of Medicine, Technion-Israel Institute of Technology, Haifa 32000, Israel; dvir.gilboa@gmail.com

**Keywords:** obesity, gender-affirming surgery, chest masculinization, complications, outcome

## Abstract

**Background:** Gender dysphoria refers to the psychological distress arising from a mismatch between an individual’s physical embodiment and their internal sense of gender. Gender-affirming mastectomies can be a pivotal component of gender affirmation for transgender, non-binary, and gender expansive individuals assigned female at birth. The impact of obesity on the outcomes of gender-affirming mastectomies has yet to be fully defined. **Methods:** A retrospective review of 205 gender-affirming mastectomies performed by the senior author was conducted. Patients were categorized into obese (BMI ≥ 30) and non-obese groups. Baseline characteristics, intraoperative variables, and complication rates were compared. Univariate and multivariate models were performed to evaluate the association between obesity and postoperative complications. **Results:** Obese patients had higher mean resection weights and liposuction volumes (*p* < 0.001). Significant differences were observed in the prevalence of fibromyalgia, prior chest surgeries, and hormone therapy usage (*p* = 0.002, 0.002, and 0.03, respectively). However, no statistically significant differences were found in overall complication rates between obese and non-obese groups in the univariate or multivariate analyses. **Conclusions:** Our study suggests that obesity is not a significant risk factor for complications in gender-affirming mastectomies patients. The varying impact of high BMI and obesity on surgical outcomes in different surgical fields highlights the importance of patient-centered care and a holistic and individual approach for each patient.

## 1. Introduction

Gender dysphoria refers to the psychological distress related to a mismatch arising from a mismatch between a individual’s physical embodiment and their internal sense of gender. This incongruence can have a damaging impact on quality of life, psychological stability, and mental health [1,2,3,4]. Timely and appropriate gender-affirming care has been shown to mitigate many of these adverse effects, leading to improved emotional well-being and reduced psychiatric comorbidities [3].

The medical armamentarium includes a wide array of surgical and non-surgical modalities to assist individuals throughout the gender-affirmation process. These interventions are commonly initiated before surgical steps are considered, so as to improve patients’ well-being and decision readiness [5,6,7].

Traditional non-surgical therapy is often the first step in the gender-affirmation process and is followed by gender-affirming surgeries of different kinds. Gender-affirming mastectomies are often regarded as a critical surgical procedure in the female-to-male transition. These procedures are geared towards creating a more masculine chest appearance [8,9]. Chest dysphoria, characterized by distress over the chest’s appearance, is a central indication for surgery and is often alleviated after mastectomy.

To improve surgical outcomes, patients are typically evaluated by a multidisciplinary committee that uses a holistic approach to determine surgical readiness, psychological stability, and adherence to medical therapy.

Obesity, commonly defined using a body mass index (BMI) threshold (≥30 kg/m^2^), is often regarded as a proxy for excess body fat, but fails to capture all aspects of body composition and metabolism. Regardless, BMI remains widely used in clinical practice and is frequently cited as a factor that may increase surgical risk. Globally, obesity is a growing concern in Western countries. Epidemiologically, obesity is a global public health challenge, with its prevalence tripling over the past five decades. According to the World Health Organization (WHO), over 1 billion people are categorized as obese, including increasing rates among adolescents [10,11]. The impact of obesity on surgical outcomes has been extensively studied. Numerous studies suggest that a higher BMI is associated with increased risk of complications, such as infection, hematoma, seroma, and impaired wound healing, in various surgical procedures [12,13,14,15]. However, regarding gender-affirming procedures specifically, the evidence is limited and mixed. Some studies report higher complication rates among patients with higher BMI, while others have found comparable outcomes regardless of BMI [16,17,18,19,20]. Many of these studies are constrained by small sample sizes, heterogeneous techniques, or lack of surgeon-specific controls.

BMI thresholds are still used as exclusion criteria for gender-affirming surgery eligibility in some settings, restricting access to medically necessary care despite limited proven data to support. This highlights the need for robust evidence to support patient selection and improve outcomes and access to care.

In this study, we aim to present a single surgeon’s experience with gender-affirming mastectomies and investigate whether obesity impacts surgical outcomes. By controlling for operator-dependent variables, we will provide a standardized analysis of obesity’s effect in this surgical field.

## 2. Materials and Methods

### 2.1. Ethical Considerations

This study was conducted after IRB approval was granted. All researchers adhered meticulously to the approved research protocol, including data de-identification and secure storage. Given the retrospective nature, informed consent was waived per IRB guidance. In any instances of ethical queries, the local IRB was consulted.

### 2.2. Data Collection

Surgical records of all 300 patients who underwent gender-affirming mastectomies at the senior author’s (Y.W.) clinic during the defined study period (2003–2023) were reviewed to determine relevance for inclusion. Patients were included if they had undergone gender-affirming mastectomy during the study period, adhered to postoperative follow-up of at least 90 days, and had BMI measurements documented.

Patients with a BMI ≥ 30 were categorized as obese. Demographic data at the time of surgery included age, BMI, comorbidities (such as hypertension, diabetes mellitus, asthma, and hypothyroidism), smoking status, prior chest surgeries, and hormone therapy regimen. Intraoperative data such as surgical technique, flap resection weight, and liposuction volume were extracted. Postoperative outcomes included hematoma, seroma, surgical site infection (SSI), dehiscence, and scarring.

### 2.3. Surgical Technique

This trial describes our experience with four main surgical approaches: the peri-areolar approach, the omega-shaped resection (nipple−areola complex on scar), the spindle-shaped mastectomy with inferior nipple−areola complex (NAC) flap, and the spindle-shaped mastectomy with a free NAC graft.

The peri-areola approach involves cutting around and reducing the areola, followed by the creation of a dermal flap to support the nipple−areola complex. The breast tissue is dissected through this incision, and redundant skin is tightened by concentric suturing.

Omega-shaped resection extends the incision medially and laterally into an omega (Ω) shape. This allows for broader resection of the skin and glandular tissue. Closure results in a horizontal scar at the level of the nipple, facilitating improved contouring in patients with moderate excess skin.

The spindle-shaped mastectomy with inferior NAC flap leaves a 2 mm thick inferiorly based skin flap carrying the NAC. A round opening is created in the superior skin flap at the ideal nipple position, and the NAC is inset through this window. The spindle-shaped excision is then closed with a horizontal scar typically placed 1–2 cm below the new NAC.

In the free NAC technique, the entire NAC is removed as a full-thickness graft following a complete spindle mastectomy. After de-epithelialization of the graft site, the NAC is repositioned and secured as a free skin graft. This method is often preferred in patients with significant breast size or ptosis.

### 2.4. Statistical Analysis

Statistical analysis was carried out using the software Statistical Package for Social Sciences (SPSS version 29.0). Categorical variables were described as frequencies of the total group, while continuous variables were presented as mean and standard deviations. The comparison of categorical variables was conducted using the Chi-square test or Fisher’s exact test when the number of cases in a cell was less than 5. Continuous variables were compared using the independent Samples *t*-test or Wilcoxon’s ranked sum test, if not normally distributed. Bonferroni correction was used to account for multiple comparisons in relevant analyses. Logistic regression was employed for further analysis, with adjustments made for potential confounding variables that differ between the study groups and are suspected to impact complication rates in gender-affirming mastectomies.

## 3. Results

In total, 205 patients met the inclusion criteria and were enrolled in the study. Among these, 35 patients (17%) had body mass index (BMI) values indicative of obesity (BMI ≥ 30), while the remaining 170 patients comprised the non-obese group. All patients completed the required minimum postoperative follow-up period of 90 days, and complete datasets were available for all of the included variables.

Baseline demographic and clinical characteristics were compared between the two groups. While most variables were similar, three key differences reached statistical significance. Fibromyalgia was more commonly recorded in the medical files of obese patients (5.7% vs. 0%; *p* = 0.002), as well as prior chest surgeries (8.6% vs. 0.6%; *p* = 0.002). Furthermore, a combination of gender-affirming hormone therapy (GAHT) and estrogen blockade were used more frequently in the obese cohort (31.4% vs. 15.9%; *p* = 0.03). Detailed stratification based on these variables is shown in Table 1.

Intraoperative findings revealed substantial differences in resection weights and liposuction volumes. Obese patients underwent procedures with significantly higher average resection weights (850.5 ± 472.2 g vs. 424.5 ± 276.3 g; *p* < 0.001) and greater mean liposuction volumes (469.6 ± 471.0 mL vs. 146.8 ± 197.6 mL; *p* < 0.001). Additionally, there was a statistically significant difference in the distribution of the. surgical techniques employed, with “Free NAC” more commonly used among obese patients (*p* = 0.04). Full surgical variable comparisons are shown in Table 2.

When comparing postoperative outcomes, univariate analysis revealed no statistically significant differences in the incidence of complications between the obese and non-obese groups. Specifically, rates of surgical site infection (SSI), thromboembolism, wound dehiscence, hypertrophic scarring, seroma, and hematoma were comparable. Notably, hematoma occurred in only one patient in the obese group versus 19 patients in the non-obese group, although this difference did not reach statistical significance (*p* = 0.13). Detailed outcomes are provided in Table 3.

Stratification of surgical techniques by obesity status demonstrated no statistically significant differences in the rate of the various complications. The only exception was a higher prevalence of wound dehiscence observed among non-obese patients who underwent the NAC flap technique (*p* < 0.001), as shown in Table 4.

To further explore potential confounding effects and assess independent predictors of complications, multivariate logistic regression models were constructed. Variables significantly associated with obesity in earlier analyses—namely, previous chest surgery, fibromyalgia, and combined GAHT plus estrogen blockade—were included in the adjusted models. After adjustment, obesity was not found to be a statistically significant predictor for any complications, including seroma, hematoma, dehiscence, or hypertrophic scarring. Odds ratios and confidence intervals are presented in Table 5.

## 4. Discussion

In this large single-surgeon cohort, we found no statistically significant difference in surgical outcomes between obese and non-obese patients undergoing gender-affirming mastectomies. Adjustment to potential confounders in a multivariate logistic regression model reinforced the conclusion that BMI is not an independent predictor of post-operative complications. Importantly, at baseline, the groups did not differ in health status and comorbidities, minimizing the likelihood of bias stemming from hidden confounders.

The findings from our cohort are consistent with previous research on gender-affirming surgery outcomes. As presented previously, numerous studies demonstrated BMI does not significantly impact complication rates, post-operative satisfaction, or body image in patients following gender-affirming mastectomies [18,19,20]. Synthesizing with our results, the growing body of evidence suggests that, unlike in many other surgical procedures, gender-affirming mastectomies are safe regardless of obesity status, and it should not limit candidacy for surgery.

This conclusion contrasts the general plastic surgery literature, where obesity is a well-established risk factor for post-operative complications. In a large systematic review, Biagrella et al. [21] reported that obese patients have a 62% increased risk of complications across the entire spectrum of plastic surgery procedures. Despite a lack of definite evidence to support hypotheses on potential explanations for the discrepancy, one possible explanation can be that patients seeking gender-affirming mastectomies are generally younger; more medically optimized; and highly motivated to adhere to postoperative recommendations such as wound care, smoking cessation and lifestyle modifications. These factors may, in part, mitigate the potential risk associated with obesity in surgical patients.

Given the consistent evidence regarding the safety of gender-affirming mastectomies in patients with BMI > 30, we advocate that BMI alone should not be used as a strict exclusion criterion for surgical candidacy. Maintaining BMI-based restriction may unnecessarily delay access to medically necessary care, exacerbate gender dysphoria, and contribute to significant mental health burdens. Goetz et al. [22] described the profound psychological impact on patients’ psychological well-being when gender-affirming surgery is denied based on weight-based criteria. Our findings emphasize the need for individualized, patient-centered decision-making processes that estimate overall health status and decision readiness over arbitrary BMI thresholds.

In our practice, no strict upper BMI limit exists, and the institution does not impose a mandatory cut-off in patients that were considered medically capable to undergo the procedure by the multidisciplinary treating team.

Additionally, as this study explores a single institution’s experience with gender-affirming care, practices may differ those employed at other institutions globally. One example is the low frequency of patients receiving pre-operative testosterone GAHT, compared to the USA. Possible explanations include regional practices, guideline differences, or patient preference. In our institutions, documented history of GAHT was not a prerequisite for surgical candidacy, which is often the case in other countries.

It is crucial to mention the limitations associated with our presented research. The retrospective study design and the single-surgeon, single-institution setting may limit generalizability of our findings. Furthermore, only 17% of the study population had a BMI ≥ 30, this alongside the limited sample size may limit the statistical power that is necessary to detect subtle differences in complication rates. These limitations underline the need for ongoing large-scale research to strengthen the evidence base and to draw definite conclusions. Although randomized-controlled trials are neither feasible nor ethical for this research question, large-scale multi-institutional prospective studies and registry analyses can validate, as best as possible, our results.

The integration of patient-reported outcome measures (PROMs) and qualitative studies can also further broaden our understanding of the topic and add to the much needed and valuable discussion on the consequences of limiting access to care and surgical denial.

In conclusion, the results presented in this study add to the substantial body of evidence that obesity does not impair surgical outcomes in gender-affirming mastectomies, and should not limit surgical candidacy. Informed decision-making and a holistic overview of patients and their surgical risk must replace arbitrary BMI thresholds, to help ensure equitable access to medical care and surgical safety.

## Figures and Tables

**Table 1:** Univariate analysis focusing on differences in baseline demographic and medical characteristics between obese and non-obese patients.

Variable	Patients with Obesity N = 35	Patients Without Obesity N = 170	*p* Value
Age, mean ± SD.	21.5 ± 7.6	20.6 ± 6.5	0.51
BMI, mean ± SD.	34.9 ± 4	23.6 ± 3.4	<0.001
Hypertension	0 (0%)	1 (0.6%)	0.65
Diabetes mellitus	1 (2.9%)	2 (1.18%)	0.45
Fibromyalgia	2 (5.7%)	0 (0%)	0.002
Asthma	4 (11.4%)	5 (2.9%)	0.03
GAHT	16 (45.7%)	74 (43.5%)	0.81
Estrogen blockers	1 (2.9%)	5 (2.9%)	0.98
GAHT + Estrogen blockers	11 (31.4%)	27 (15.9%)	0.03
Previous chest surgery	3 (8.6%)	1 (0.6%)	0.002

**Table 2:** Univariate analysis focusing on differences in surgical characteristics between obese and non-obese patients.

Variable	Obese Patients (N = 35)	Non-Obese Patients (N = 170)	*p* Value
Surgical technique:			0.04
Peri-areolar	0 (0%)	18 (10.6%)	
NAC on scar	0 (0%)	4 (2.3%)	
NAC flap	4 (11.4%)	37 (21.8%)	
Free NAC	31 (88.6%)	110 (6.5%)	
Average resection weight (g), mean ± SD.	850.5 ± 472.2	424.5 ± 276.3	<0.001
Average liposuction volume (mL), mean ± SD.	469.6 ±471.0	146.8 ± 197.6	<0.001

**Table 3:** Univariate analysis focusing on differences in frequency of post-operative complications in obese patients compared to non-obese patients.

Variable	Obese Patients (N = 35)	Non-Obese Patients (N = 170)	*p* Value
SSI	0 (0%)	1 (0.59%)	0.65
Thromboembolism	0 (0%)	0 (0%)	1
Wound Dehiscence	4 (11.4%)	39 (22.9%)	0.13
Hypertrophic scarring	4 (11.4%)	9 (5.3%)	0.18
Seroma	5 (11.4%)	14 (8.3%)	0.27
Hematoma	1 (11.4%)	19 (11.2%)	0.13
Return to operation room	0	1 (0.59%)	0.65

**Table 4:** Stratification of surgical techniques by obesity status and prevalence of complications in each group.

	Non-Obese (N = 170)	Obese (N = 35)	*p* Value
Hypertrophic Scars:			1
Peri-areolar.	0	0	
NAC on Scar.	1	0	
NAC Flap.	1	0	
Free NAC.	7	4	
Seroma:			1
Peri-areolar.	1	0	
NAC on Scar.	4	0	
NAC Flap.	2	0	
Free NAC.	7	9	
Hematoma:			1
Peri-areolar.	14	0	
NAC on Scar.	0	0	
NAC Flap.	0	0	
Free NAC.	5	1	
Wound Dehiscence:			<0.001
Peri-areolar.	0	0	
NAC on Scar.	0	0	
NAC Flap.	37	4	
Free NAC.	2	0	
Surgical Site Infection:			1
Peri-areolar.	0	0	
NAC on Scar.	1	0	
NAC Flap.	0	0	
Free NAC.	0	0	

**Table 5:** Multivariate logistic regression models predicting the risk for complication development in obese patients. Results are adjusted for surgical technique, previous chest surgery, and a combination of GAHT and hormone blockers.

Complication	Odds Ratio (95% Confidence Interval)
Seroma	1.03 (0.12–8.56)
Hematoma	1
SSI	1
Wound dehiscence	1
Hypertrophic scarring	1.8 (0.16–21.4)

## Data Availability

The original contributions presented in this study are included in the article. Further inquiries can be directed to the corresponding author(s).
